# Landmark Data to Distinguish and Identify Morphologically Close *Tabanus* spp. (Diptera: Tabanidae)

**DOI:** 10.3390/insects12110974

**Published:** 2021-10-28

**Authors:** Tanasak Changbunjong, Nutnicha Prakaikowit, Photchanun Maneephan, Tipparat Kaewwiset, Thekhawet Weluwanarak, Tanawat Chaiphongpachara, Jean-Pierre Dujardin

**Affiliations:** 1 Department of Pre-Clinic and Applied Animal Science, Faculty of Veterinary Science, Mahidol University, Nakhon Pathom 73170, Thailand; nutnicha.pra@student.mahidol.edu (N.P.); photchanun.man@student.mahidol.edu (P.M.); tipparat.kea@student.mahidol.edu (T.K.); 2 The Monitoring and Surveillance Center for Zoonotic Diseases in Wildlife and Exotic Animals (MoZWE), Faculty of Veterinary Science, Mahidol University, Nakhon Pathom 73170, Thailand; thekhawet.wel@mahidol.edu; 3 Department of Public Health and Health Promotion, College of Allied Health Science, Suan Sunandha Rajabhat University, Samut Songkhram 75000, Thailand; tanawat.ch@ssru.ac.th; 4 Institut de Recherche pour le Développement (IRD), Unité Mixte de Recherches INTERTRYP (IRD, et Centre de Coopération Internationale en Recherche Agronomique pour le Développement, CIRAD), University of Montpellier, F-34398 Montpellier, France; dujjepi@gmail.com

**Keywords:** geometric morphometrics, horse flies, *Tabanus megalops*, *Tabanus rubidus*, *Tabanus striatus*, vector

## Abstract

**Simple Summary:**

*Tabanus* spp. (Diptera: Tabanidae) are blood-sucking parasites of animals and humans. The accurate identification of these flies is very important for determining the vector species involved in disease transmission and for planning effective vector control and management strategies. We explored the effectiveness of landmark-based geometrics at distinguishing and identifying morphologically similar species of *Tabanus* (*T. megalops*, *T. rubidus*, and *T. striatus*) in Thailand. Our study reveals that geometric morphometrics is effective at distinguishing between the three species of *Tabanus*. Furthermore, our study material can be used as reference material for species identification.

**Abstract:**

*Tabanus* spp., also known as horse flies (Diptera: Tabanidae), are important vectors of several animal pathogens. Adult females of *Tabanus* *megalops* and *Tabanus striatus*, which are members of the *T. striatus* complex, are morphologically similar and hence difficult to distinguish using morphological characteristics. In addition, molecular identification by DNA barcoding is also unable to distinguish these species. These two species can occur sympatrically with *Tabanus rubidus*, which is morphologically similar to *T. megalops* and *T. striatus*. Wing geometric morphometrics has been widely used in various insects to distinguish morphologically similar species. This study explored the effectiveness of landmark-based geometrics at distinguishing and identifying *T. megalops*, *T. rubidus*, and *T. striatus* in Thailand. Specimens were collected from different geographical regions of Thailand, and only unambiguously identified specimens were used for geometric morphometric analyses. Left wings of females of *T. megalops* (n = 160), *T. rubidus* (n = 165), and *T. striatus* (n = 85) were photographed, and 22 wing landmarks were used for the analysis. Wing shape was able to distinguish among species with high accuracy scores, ranging from 94.38% to 99.39%. We showed that morphologically very close species of *Tabanus* can be reliably distinguished by the geometry of their wing venation, and we showed how our experimental material could be used as a reference to tentatively identify new field collected specimens.

## 1. Introduction

*Tabanus* spp., also known as horse flies (Diptera: Tabanidae), are hematophagous flies of medical and veterinary importance. They are classified into the suborder Brachycera, the infraorder Tabanomorpha, and the family Tabanidae. Approximately 1300 species have been described [1]. Female flies feed on pets, livestock, wildlife, and occasionally, humans. They are biological vectors of *Trypanosoma theileri*, and mechanical vectors of other trypanosomes, such as *Trypanosoma brucei*, *Trypanosoma congolense*, *Trypanosoma evansi*, and *Trypanosoma vivax*. Moreover, they can mechanically transmit other pathogens, such as the etiologic agents of infectious diseases like African horse sickness, anthrax, bovine anaplasmosis, bovine besnoitiosis, bovine leucosis, equine infectious anemia, lumpy skin disease, and tularemia [2,3,4]. In Thailand, approximately 80 species of *Tabanus* and their distributions have been recorded [5,6,7]. The most common species are *T. striatus*, *T. megalops*, and *T. rubidus* [5,7,8,9]. All three species were also reported in the epidemic area of trypanosomosis in Central Thailand [8].

**Figure 1:**
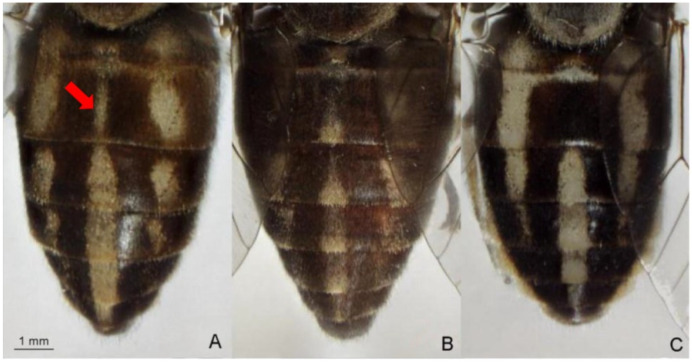
Morphological characteristics of abdominal dorsum used to distinguish species of *Tabanus megalops* (**A**), *T. rubidus* (**B**), and *T. striatus* (**C**). *Tabanus megalops* and *T. striatus* are distinguished by the midline of the 2nd tergite cross by a stripe of pale tomentum and hairs in *T. megalops* (arrow).

**Figure 2:**
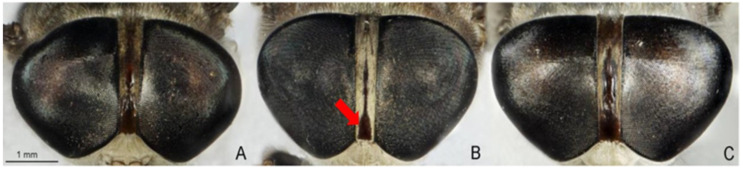
Morphological characteristics of basal callus used to distinguish species of *Tabanus megalops* (**A**), *T. rubidus* (**B**), and *T. striatus* (**C**). *Tabanus rubidus* is distinguished from *T. megalops* and *T.* *striatus* by the basal callus, which is more triangular than rectangular (arrow).

The identification of *Tabanus* spp. employs morphological and molecular methods [5,10,11,12,13,14]. Morphological identification is largely based on head structures, particularly the characters of the callus, antennae, eyes, frons, and beard, together with the color and patterns of the body, legs, and wings [5]. This method is relatively simple and economical. It does not require any complicated equipment, but requires experienced taxonomists. The specimens for this method must have clear external morphological characteristics [12]. To solve morphological problems, DNA barcoding has been widely used [10,11,12,13,14]. This method was useful for the identification of many *Tabanus* spp.; it was however, unable to distinguish the members of the *T. striatus* complex from Thailand [12]. *Tabanus striatus* and *T. megalops* are members of the *T. striatus* complex, which are found in the Oriental region [5]. Based on morphological identification, *T. striatus* and *T. megalops* are distinguished by the midline of the second tergite, crossed by a stripe of pale tomentum and hairs in *T. megalops,* which are not present in *T. striatus*. In addition, the dark pattern on the abdominal dorsum of *T. striatus* is generally darker than that of *T. megalops* [5]. Although *T. striatus* and *T. megalops* are easily distinguished using these two characteristics, when *T. megalops* is stained and/or rubbed on the second tergite, it may have a *T. striatus*-like appearance [5] (Figure 1). *Tabanus rubidus* is distinguished from the other two species by the basal callus, which is more triangular than rectangular [5] (Figure 2). The accurate identification of these species is crucial not only to recognize the vectors involved in pathogen transmission, but for the development of appropriate control strategies. Since DNA barcoding is unable to distinguish between *T. striatus* and *T. megalops*, alternative methods, such as geometric morphometrics, have become invaluable.

Wing geometric morphometrics is increasingly used for insects of medical and veterinary importance to distinguish morphologically similar species, explore intraspecific variation among populations, and determine sexual dimorphism [15,16,17,18,19,20,21,22]. The method is fast, low-cost, and easy to use [15]. Geometric morphometric analysis can be performed using various methods, such as landmark, semi-landmark, and outline-based methods, depending on the characteristics and specifics of the specimens [15,23,24]. The effectiveness of geometric morphometrics for species identification has been demonstrated in various models, including blow flies [20], flesh flies [25], mosquitoes [16,21,26,27,28,29], stomoxyine flies [18], sand flies [30,31,32], and tsetse flies [33].

In the present study, landmark-based geometric morphometrics was used to differentiate three *Tabanus* spp, namely, *T. megalops*, *T. rubidus*, and *T. striatus*, in Thailand. To generalize our results and provide an alternative method for the identification of these flies, our study material was used as reference data for the morphometric identification of additional, field collected *Tabanus* spp.

## 2. Materials and Methods

### 2.1. Ethical Statement

Our protocol for specimen collection was approved by the Faculty of Veterinary Science, Mahidol University Animal Care and Use Committee (Ref. MUVS-2020-01-01).

### 2.2. Fly Collection and Species Determination

**Figure 3:**
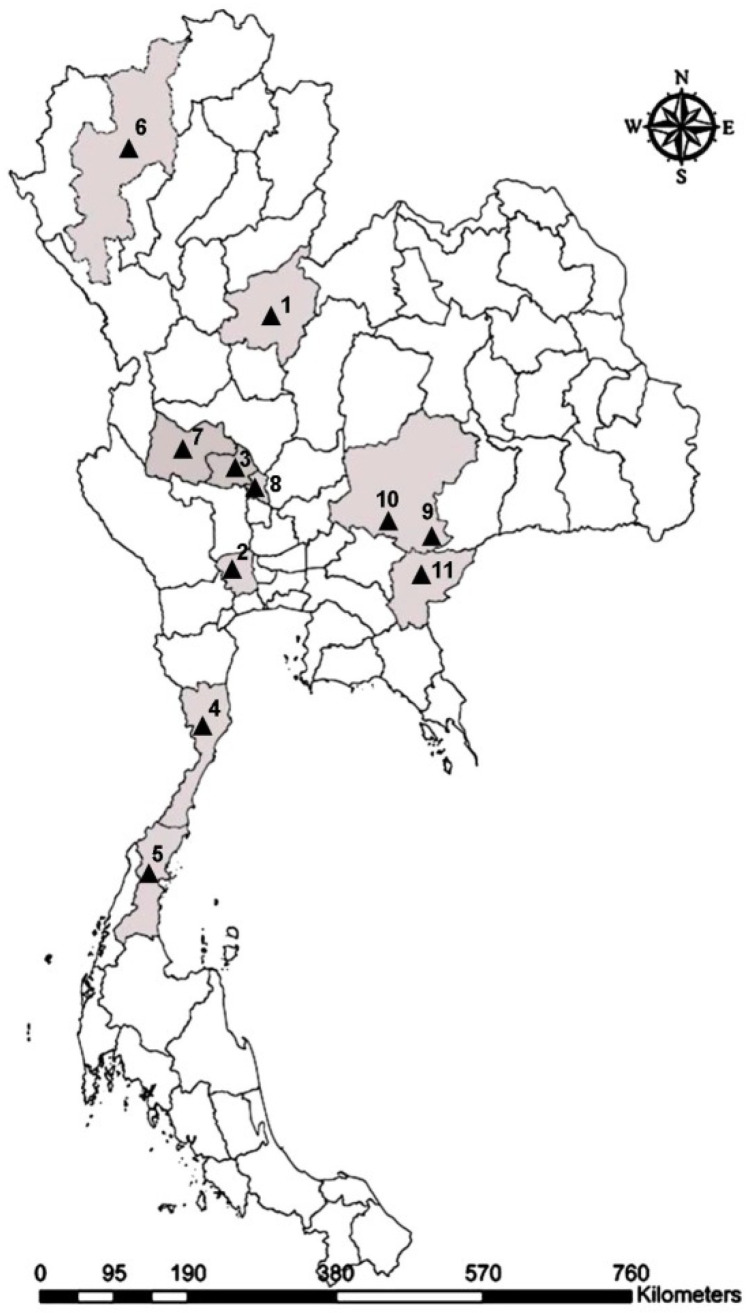
Map of *Tabanus* collection sites in Thailand: Phitsanulok (1), Nakhon Pathom (2), Chainat (3), Prachuap Khiri Khan (4), Chumphon (5), Chiang Mai (6), Uthai Thani (7), Singburi (8), Nakhon Ratchasima (9, 10), and Sa Kaeo (11).

Specimens of *T. megalops*, *T. rubidus*, and *T. striatus* were collected from different geographical regions of Thailand using five Nzi Traps [34] between February 2020 and January 2021 (Table 1, Figure 3). The traps were randomly placed at the collection sites from 06:00 to 18:00 over a two-day period. All flies were euthanized in a freezer (−10 °C) and placed in individual 1.5 mL microcentrifuge tubes. The specimens were transported to the Vector-Borne Diseases Research Unit, Faculty of Veterinary Science, Mahidol University. They were stored at −20 °C until morphological identification. Species recognition was performed on unambiguous specimens, i.e., those presenting clearly the specific traits or diagnostic characters, according to the descriptions and taxonomic keys of Burton (1978) [5].

A separate set of specimens (also known as unknown specimens) was collected from other geographic areas as a test of the robustness of morphometric analysis based on 410 reference wings to differentiate species. They included *T. megalops* (Unknown A, n = 10, from Chainat Province, Central Thailand), *T. rubidus* (Unknown B, n = 10, from Uthai Thani Province, Northern Thailand), and *T. striatus* (Unknown C, n = 10, from Nakhon Ratchasima Province, Northeastern Thailand). Most of them had clear morphological characteristics, but some specimens of *T. megalops* exhibited an unclear stripe on the second tergite.

### 2.3. Geometric Morphometric Analysis

#### 2.3.1. Wing Preparation 

The left wings of females of *T. megalops*, *T. rubidus*, and *T. striatus* were dissected from their bodies and carefully mounted with Hoyer’s medium on microscope slides. All wing slides were photographed using a digital camera connected to a stereomicroscope (Nikon AZ 100, Nikon Corp, Tokyo, Japan) and a scale bar attached to prevent errors in the sizing of each wing. A total of 410 wing images, comprising 160 wings of *T. megalops*, 165 wings of *T. rubidus*, and 85 wings of *T. striatus*, were analyzed using the landmark-based geometric morphometric method (Table 1 and Table S1).

#### 2.3.2. Inter-User Repeatability

To check the precision of landmark digitization, a repeatability test was used for size and for shape, separately. It was computed as the ratio of the variance due to differences among individuals to the total variance [35]. The computation of the variance components of shape followed the Procrustes ANOVA method proposed by Klingenberg and Mclntyre (1998) [36]. Ten wings per species were randomly selected and digitized between two different users. If the repeatability value (of shape) was less than 0.9, all wing pictures were re-digitized.

#### 2.3.3. Landmark-Based Analysis

**Figure 4:**
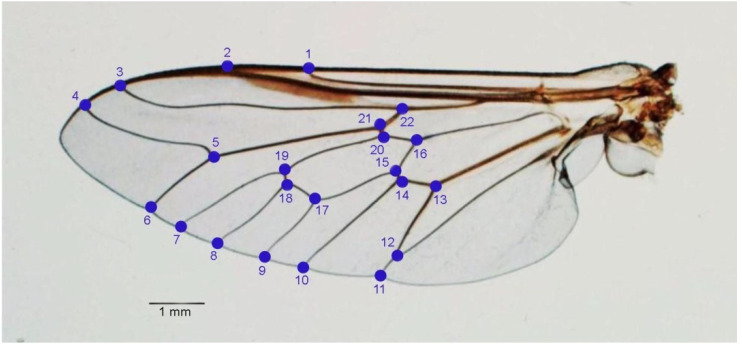
The twenty two landmarks digitized on the wing of *Tabanus* spp. for landmark-based geometric morphometric analysis.

Coordinates of 22 wing landmarks (Figure 4) were digitized for geometric morphometric analysis. To show size variation among the groups, the global wing size was estimated using the centroid size (CS) derived from the coordinates of all landmarks. The CS is defined as the square root of the sum of the squared distances between the centroid and each landmark [37]. Statistical comparisons of the CS among the species were performed by one-way ANOVA and illustrated by quantile boxes. The statistical significance of the one-way ANOVA was estimated by a non-parametric procedure (1000 permutations) at *p*-value < 0.05.

The wing shape variables were computed after a Procrustes superimposition according to the Generalized Procrustes Analysis (GPA, see Rohlf (1990) [38]). Their principal components were used as final shape variables. The visual comparison of shape changes across species was provided by the superposition of the average wing of each species. The final wing shape variables (thus, excluding size) were used as input for discriminant analysis (DA), which were illustrated by the factor map. Statistical significance of the Mahalanobis distances among the species was estimated by a non-parametric permutation test (122 permutations) at *p*-value < 0.05.

#### 2.3.4. Classification Based on Size and Shape

To test the validity of global size (CS) for accurate species identification, we used a maximum likelihood approach [39]. To measure the taxonomic signal embedded in the shape variables, excluding size, we used a discriminant model. The latter is based on the shortest Mahalanobis distance between each specimen and the consensus shape of each species. Both classifications (size, shape) were validated classifications. Thus, each individual was sequentially removed from the total sample and assigned to the most likely (size) or closest (shape) group, without being used to aid the computation of the model (jack-knife classification; see Manly (2004) [40]).

#### 2.3.5. Allometric Effect Analysis

The allometric effect (the effect of size on shape variation) was performed by linear regression of the first (shape derived) discriminant factor on the CS, and then estimated by the determination coefficient r^2^. Thus, in our study the estimation of allometry focused on the between species shape-based discrimination.

#### 2.3.6. Identification of Unknown Specimens in the Field

Our study material was used as reference data to determine 30 additional field collected specimens. These specimens came from different areas, and were used as testing specimens to be identified using the three reference groups of our study (*T. megalops*, *T. rubidus*, and *T. striatus*).

The identification algorithm used the shortest Mahalanobis distance of each specimen to the mean shape of each species in the same way as performed for a cross-validated classification: each unknown specimen used the discriminant space of the reference data but did not contribute to its computation [41]. Due to the necessity to recompute shape variables before each individual assignment, the repeatedly built discriminant functions are never exactly the same. This “one by one” identification process has been explained in Dujardin et al. (2010) [41] and applied in Kitthawee and Dujardin (2016) [42]. For each individual, the final species attribution was decided according to its shortest Mahalanobis distance with one of the three reference groups.

A hierarchical clustering tree (UPGMA algorithm) based on the Mahalanobis distances among the average wing shapes was used to illustrate the relationships between reference data and unknown specimens. Branch support was estimated based on 1000 bootstrap replicates of the data [43,44].

#### 2.3.7. Morphometric Software

Geometric and multivariate analyses were performed using XYOM (XY Online Morphometrics) version 2 software [24], freely accessible at https://xyom.io/, accessed on 16 October 2021.

## 3. Results

### 3.1. Inter-User Repeatability

The two sets of measurements performed by two different users on the same images yielded high repeatability scores: 98% for size and 93% for shape.

### 3.2. Wing Size Variation

**Figure 5:**
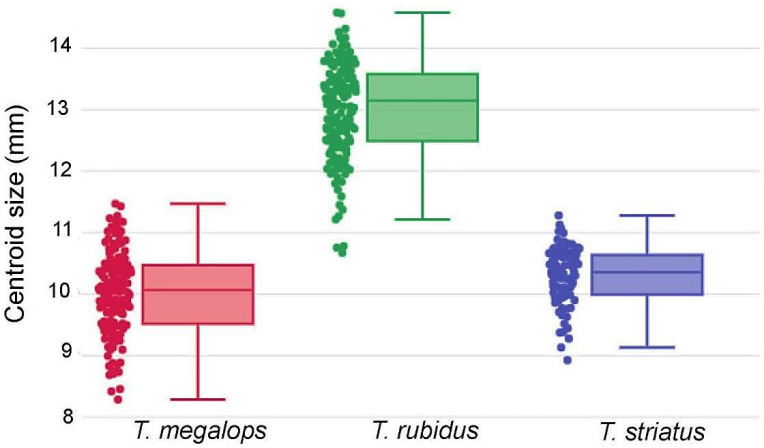
Quantile boxes of centroid size variations of *Tabanus megalops* (red, leftmost box), *T. rubidus* (green, central box), and *T. striatus* (blue, rightmost box). The horizontal line crossing each box is the median separating the 25th and 75th quartiles.

The variations in wing size (CS) among *Tabanus* spp. are illustrated by quantile boxes (Figure 5). The largest wings were found in *T. rubidus* (13.01 ± 0.77 mm), followed by *T. megalops* (10.01 ± 0.67 mm) and *T. striatus* (10.29 ± 0.47 mm). Only the size of *T. rubidus* was significantly different from the two other species (*p* < 0.05). The accuracy of the maximum likelihood validated size-based classification was very high for *T. rubidus* (95.76%), but less satisfactory for *T. megalops* (66.25%) and *T. striatus* (56.65%).

### 3.3. Wing Shape Variation

**Figure 6:**
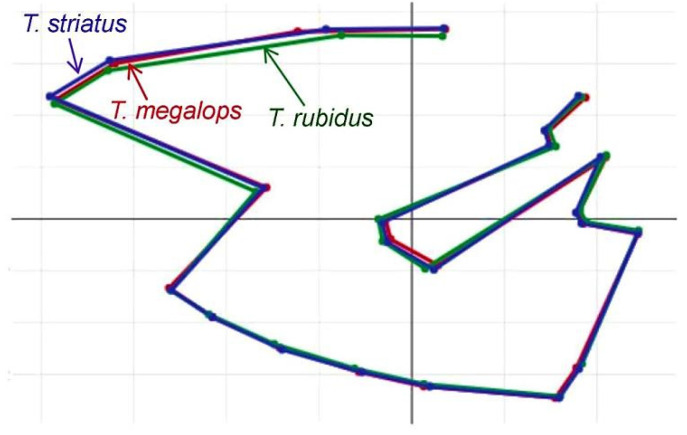
Mean anatomical landmark positions of *Tabanus megalops* (red), *T. rubidus* (green), and *T. striatus* (blue) after Procrustes superposition.

**Figure 7:**
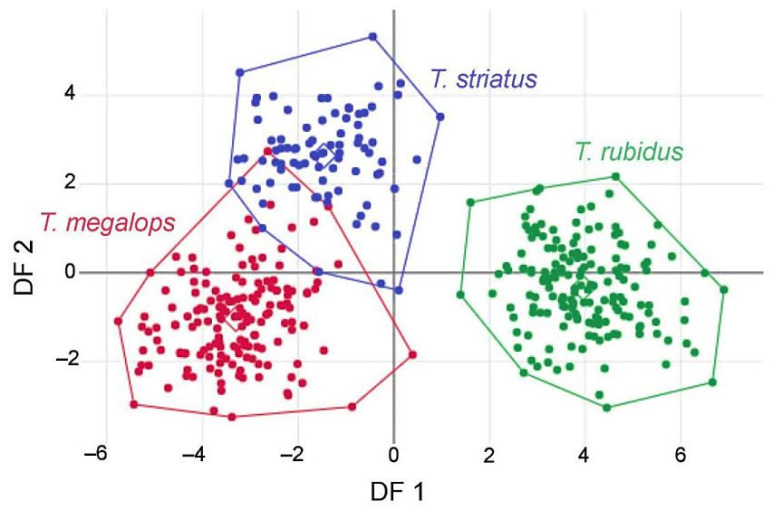
Factor map of the discriminant factors (DF) showing shape divergence of *Tabanus megalops* (red), *T. rubidus* (green), and *T. striatus* (blue). Together, the two DF represent 100% of the total discriminant space: 85% for DF1 (horizontal axis) and 15% for DF2 (vertical axis).

The visual comparisons of superposed configurations (Figure 6) revealed most visible landmark displacement in the anterior and middle part of the wing. The interspecific Mahalanobis distances between wing shapes were statistically significant (Table 2). The plot of the individuals on the two shape-derived discriminant factors showed *T. rubidus* specimens as a clear-cut external group, whereas *T. megalops* and *T. striatus* showed some overlap (Figure 7). The accuracy scores, after validated classification, were in the range of 94.38–99.39% (Table 3).

### 3.4. Allometric Effect

**Figure 8:**
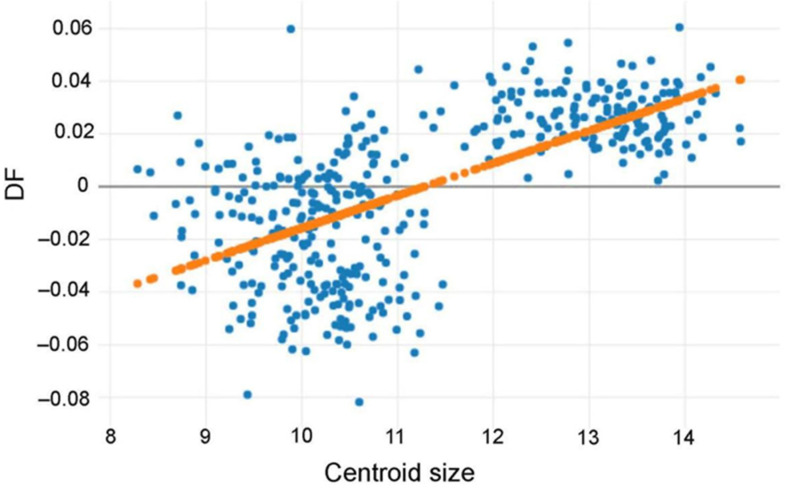
Linear regression prediction (orange dots line) of the first shape-based discriminant factor (DF) (vertical axis) according to the centroid size variation (horizontal axis).

The shape-based discrimination between species was significantly (*p* < 0.001) influenced by size (r^2^ = 43%) (Figure 8).

### 3.5. Identification of Unknown Specimens in the Field

The 30 field specimens were collected from other geographic areas. They were treated as “unknown” specimens to be compared to our study material, composed of *T. megalops* (n = 160), *T. rubidus* (n = 165), and *T. striatus* (n = 85).

**Figure 9:**
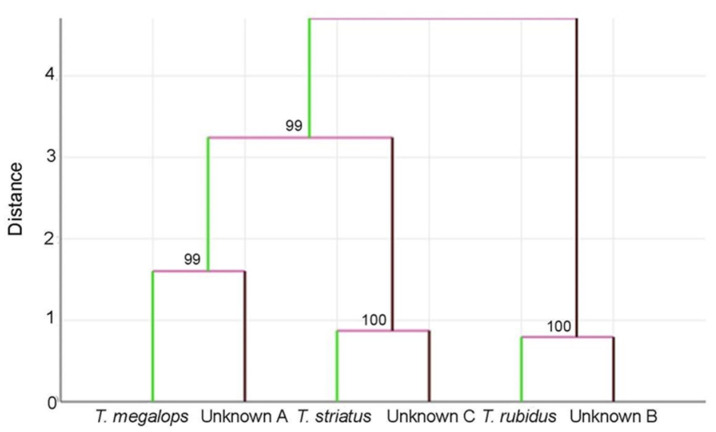
Hierarchical clustering tree based on shape similarities of reference data and unknown specimens. Unknown A: specimens from Chainat Province; Unknown B: specimens from Uthai Thani Province; and Unknown C: specimens from Nakhon Ratchasima Province. In this total sample classification analysis, all the specimens, including the unknown ones, helped with computing the discriminant model. The distances used for the tree construction were the Mahalanobis distances. Numbers at the nodes indicate the percentages of bootstrap values based on 1000 replicates.

The total sample classification, including reference and unknown groups, is illustrated by an UPGMA tree based on the relative Mahalanobis distances (Figure 9). This hierarchical clustering tree may be considered as an illustration of the global similarities between groups, but does not represent a true identification method.

The identification method was indeed performed individually for each unknown specimen (see the “one by one” method [41], a process which is difficult to illustrate graphically (see [42]). According to this method, the three groups corresponded to three different species, in agreement with the external morphological examination.

## 4. Discussion

The three *Tabanus* spp. collected for our study have been reported as the most common species in Thailand [5,7]. Some *Tabanus* spp. are morphologically similar, and hence difficult to distinguish using morphological characteristics alone. Although molecular identification methods, such as DNA barcoding, were effective in separating many *Tabanus* spp., they were unable to distinguish members of the *T. striatus* complex from Thailand [12].

Our study explored an alternative method for the identification of *Tabanus* spp. based on the geometry of their wing venation. As the evaluation of the taxonomic power of the method was based on a non-morphometric species determination, our study material was composed of specimens unambiguously identified by morphological traits. We examined successively the size and the shape of wings as derived from the Generalized Procrustes method (GPA).

The wing size of *T. megalops* and *T. striatus* was not significantly different, but consistently smaller than that of *T. rubidus*: this suggested that the wing size could aid to distinguish *T. rubidus* from *T. megalops* and *T. striatus*. In spite of the interesting reclassifications scores based on size only, we do not recommend its use as a reliable discrimination feature. Even if reflecting genetic differences, wing size can be strongly modulated by environmental factors such as temperature, relative humidity, larval density, and food availability [45,46,47].

This is why the subsequent analyses were performed excluding size, using shape variables only. However, as shown by our allometric study (Figure 8), the discriminant space based on shape was still affected by size variation. Such influence was due mainly to the presence of a relatively large species (*T. rubidus*, see Figure 5), and it did not necessarily mean that shape variation was under the influence of environmental factors [15,48]. Size divergence between species is likely to be due also to evolutionary divergence [12]. 

The discriminant space of shape (Figure 7), the quantile boxes of size (Figure 5) and the hierarchical clustering tree of total sample analysis (Figure 9) showed that *T. rubidus* was clearly separated from *T. megalops* and *T. striatus*. This was in complete agreement with the known phylogenetic relationship based on cytochrome c oxidase subunit I (COI) [12]. It was also in accordance with the morphological classification: *T. rubidus* can be clearly distinguished from *T. megalops* and *T. striatus,* i.e., the basal callus being more triangular [5].

*Tabanus megalops* and *T. striatus* are morphologically very close species, having similar size (Figure 5) and have occasionally been misidentified, especially due to the stained or rubbed off stripe on the second tergite of *T. megalops* [5]. Moreover, the COI sequences did reveal between them some overlap of intraspecific and interspecific divergence [12]. Not unexpectedly, as can be visualized by their partial overlapping in the discriminant space (Figure 7), the geometry of their wing venation could not recognize them perfectly (94%, 95%, versus 99% for *T. rubidus*).

However, the use of the 410 images to classify images of individuals coming from other geographic locations provided satisfactory results. It gave us confidence in their possible use as a reference dataset for additional identifications, even of a single individual. The use of reference images to allow geometric morphometrics to be applied as a taxonomic tool is an old idea [44,49,50]. Its application can be made difficult by measurement error. When different observers digitize the same images, repeatability does not reach the level obtained when repeated measurements are performed by the same observer. This might be particularly problematic when the objective is to recognize morphologically very similar or cryptic species [43]. As an additional problem, some groups of insects may be more difficult to digitize than others. For instance, mosquitoes have wings covered by scales, hiding the precise junctions of veins. We showed that the digitization of *Tabanus* wings provided excellent precision, even with two different users, such that the material from our study can serve as a reference set of data for species identification. 

## 5. Conclusions

The accuracy of species identification is very important for determining the role of each vector in disease transmission and for planning effective vector control and management strategies. Our study revealed that landmark-based geometric morphometrics of the wing can distinguish species within the *Tabanus striatus* complex (*T. megalops* and *T. striatus*) and can distinguish this group from other similar species (*T. rubidus*). Furthermore, our tests suggest that our study material represented a robust reference dataset able to help species determination of specimens coming from other geographic areas. The geometric method can be used as a complement to morphological identification when specimens are unclear or when there is a loss of important distinguishing characters. 

## Figures and Tables

**Table 1:** Collection sites and number (N) of wing images of *Tabanus* spp. used for the landmark-based geometric morphometric analysis. Figures into brackets refer to sites in Figure 3.

Species	Regions	District/Provinces	Hosts	N
*T. megalops*	Northern	Mueang, Phitsanulok (1)	Horse and buffalo	50
	Central	Mueang, Nakhon Pathom (2)	Beef cattle and buffalo	20
		Sankhaburi, Chainat (3)	Beef cattle	10 *
	Western	Sam Roi Yot, Prachuap Khiri Khan (4)	Beef cattle	40
	Southern	Mueang, Chumphon (5)	Beef cattle	50
*T. rubidus*	Northern	Mueang, Chiang Mai (6)	Beef cattle	20
		Mueang, Uthai Thani (7)	Buffalo	10 *
	Central	Mueang, Singburi (8)	Beef cattle	45
	Northeastern	Soeng Sang, Nakhon Ratchasima (9)	Buffalo	50
	Southern	Mueang, Chumphon (5)	Beef cattle	50
*T. striatus*	Northeastern	Soeng Sang, Nakhon Ratchasima (9)	Buffalo	55
		Wang Nam Khiao, Nakhon Ratchasima (10)	Buffalo	10 *
	Eastern	Watthana Nakhon, Sa Kaeo (11)	Buffalo	30
Total				440

*, unknown specimens.

**Table 2:** Mahalanobis distances among the wing shapes of *Tabanus megalops*, *T. rubidus*, and *T. striatus*.

Species	*T. megalops*	*T. rubidus*	*T. striatus*
*T. megalops*	-		
*T. rubidus*	7.29	-	
*T. striatus*	4.12	6.19	-

**Table 3:** Validated classification based on the wing shapes of *Tabanus megalops*, *T. rubidus*, and *T. striatus*.

Species	Accuracy (Assigned/Observed)
*T. megalops*	94.38% (151/160)
*T. rubidus*	99.39% (164/165)
*T. striatus*	95.29% (81/85)
Total performance	96.59% (396/410)

## Data Availability

The data presented in this study are available in Supplementary Materials.

## Supplementary Materials

The following are available online at https://www.mdpi.com/article/10.3390/insects12110974/s1, Table S1: Raw coordinates of wing landmarks of *Tabanus megalops* (No. 1–160), *T. rubidus* (number 161–325) and *T. striatus* (number 326–410).
